# Investigating the Aging Behavior of High-Density Polyethylene and Polyketone in a Liquid Organic Hydrogen Carrier

**DOI:** 10.3390/polym15224410

**Published:** 2023-11-15

**Authors:** Jyothsna Surisetty, Mohammadhossein Sharifian, Thomas Lucyshyn, Clemens Holzer

**Affiliations:** 1 Polymer Processing, Department of Polymer Engineering and Science, Montanuniversität Leoben, 8700 Leoben, Austria; thomas.lucyshyn@unileoben.ac.at (T.L.); clemens.holzer@unileoben.ac.at (C.H.); 2 Chemistry of Polymeric Materials, Department of Polymer Engineering and Science, Montanuniversität Leoben, 8700 Leoben, Austria; mohammadhossein.sharifian@unileoben.ac.at

**Keywords:** LOHC (perhydro-benzyltoluene), POK, aging behavior, fuel uptake, long-term storage

## Abstract

Hydrogen is recognized as a significant potential energy source and energy carrier for the future. On the one hand, storing hydrogen is a challenging task due to its low volumetric density, on the other hand, a particular type of hydrogen in the form of a liquid can be used to store large quantities of hydrogen at ambient conditions in thermoplastic tanks. But storing hydrogen in this form for a long time in polymer tanks affects the physical and chemical properties of the liner. In the current automotive industry, high-density polyethylene (HDPE) has already been used in existing fuel tank applications. However long-term exposure to fuels leads to the permeation of hydrocarbons into the polymers, resulting in a loss of mechanical properties and reducing the efficiency of fuel cells (FC) in automotive applications. Additionally, facing material shortages and a limited supply of resin leads to an increase in the cost of the material. Therefore, an alternative material is being searched for, especially for hydrogen fuel tank applications. In this study, two semi-crystalline thermoplastics, HDPE and polyketone (POK), were compared, which were exposed to a selected liquid organic hydrogen carrier (LOHC) at 25 °C and 60 °C for up to 500 h in an enclosed chamber, to measure their fuel up-take. A short analysis was carried out using differential scanning calorimetry (DSC), thermogravimetric analysis (TGA), Fourier transform infrared spectroscopy (FTIR), and mechanical testing to understand the influence of the LOHC on the polymer over time. Fuel sorption and tensile properties showed a plasticizing effect on HDPE. The material degradation was more pronounced for the aged samples of HDPE in comparison to POK. As expected, thermal aging was increased at 60 °C. The fuel absorption of POK was lower compared to HDPE. A slight increase in crystallinity was observed in POK due to the aging process that led to changes in mechanical properties. Both HDPE and POK samples did not show any chemical changes during the aging process in the oven at 25 °C and 60 °C.

## 1. Introduction

Nowadays, many European countries have set their targets to attain zero emissions by 2050. An increasing awareness of environmental issues and the drawbacks of using fossil fuel have led to a significant progress in the development of hydrogen as an alternative fuel for the transportation sector [1]. During the last two decades, hydrogen has emerged as a promising topic in the current market, and is gaining popularity due to its ability to provide a CO_2_-free alternative to gasoline or diesel [1,2]. Moreover, hydrogen can be produced from renewable sources such as solar, wind, and hydroelectric power through the electrolysis of water, making it a potentially sustainable energy source [3,4]. So far, the companies Toyota, Hyundai, General Motors, and BMW have successfully launched hydrogen fuel cell vehicles in the current market, which are approved globally [5,6,7,8]. But storing the hydrogen in gaseous and liquid form in automotive applications is challenging due to its low volumetric energy density 5.6 MJ/L (less than gasoline 32 MJ/L), high pressures (700 bar), and low temperatures (−253 °C) which are difficult to handle. High production cost and storage in fuel tanks are challenges that need to be tackled [9,10]. To solve these problems, some researchers suggest storing hydrogen in the form of an organic liquid rather than in its gaseous state. This approach could potentially increase the gravimetric density up to 7.2% and provide a promising solution for safe and efficient storage of hydrogen at room temperatures of 25 °C, using regular polymer tanks. This liquid hydrogen in chemical form can absorb (hydrogenation) and release (dehydrogenation) hydrogen chemically under high temperatures and is known as a liquid organic hydrogen carrier (LOHC) [11,12,13,14,15].

Different types of LOHC are available in the market to store hydrogen such as 9-ethyl-perhydrocarbazole (H-12 NEC), perhydro-benzyltoluene (H12-BT), or methylcyclohexane (MCH) [12,16]. To store these special types of LOHCs, existing infrastructure is sufficient (like gasoline and diesel filling stations) compared to other forms of hydrogen. 

In this article, out of the different types of LOHCs, the focus is on storing H12-BT in HDPE and POK. One of the reasons for choosing this benzyltoluene (BT) is a recently introduced, promising approach to convert this BT into an on-board generation of electricity that can be used for mobile applications. For this purpose, a direct LOHC-based fuel cell (LOHC-DIPAFC) is being developed [17,18]. 

It generally consists of aromatic hydrocarbons with a carbon number of 12 and it exhibits diesel-like properties [19]. It should be considered that LOHC fuels are generally different from each other in terms of molecular structure, which leads to dissimilarity in solubility and diffusion into polymers [20,21]. In the case of petroleum fuels, the most commonly used polymeric material for tanks is HDPE because it can resist chemicals very well and has a low permeability; however, the diffusion coefficient of HDPE is higher due to its non-polar hydrophobic nature [22]. For example, Richaud et al. performed a series of immersion tests on biodiesel fuels to measure the sorption rate in polyethylene. These analyses confirmed that the absorption rate was increased with increasing time and reached a plateau after immersion for 139 h under ambient conditions [23]. Different grades of HDPE were also examined by Böhning et al. A decrease in tensile strength and an increase in strain percentage were observed at 60 °C. As the material is exposed to fuels for a longer time, thermoplastics undergo plasticization effects [21]. Therefore, in order to improve the barrier properties, fluorination (halogen gas) is introduced on the surface of HDPE as a post treatment process, but this alternative method can contribute to global warming because it emits green-house gases. Jianwei Zhao et al. investigated the absorption of different biodiesels in polyamide 6 for 720 h. After 720 h, the polymers did not reach the plateau state and grew linearly. It was also noticed that, due to the longer exposure time in polyamide 6, the material behaved as plasticizer and changes in properties were identified [24]. Michael D. Kass et al. tested immersion tests on different commodity and high-performance polymers for 16 weeks using diesel fuels. Out of those, HDPE and PP also exhibited high swelling percentages (5–15%) when exposed due to higher solubility, resulting in change in the hardness, volume, and mass of the polymers [25]. To develop liquid storage tanks, B.F. Yousif and H. Ku investigated the behavior of aged samples (6 months) in diesel using polyester and coir fibers, and improved the interfacial adhesion and tensile properties, and also the resistance to degradation when compared to glass fibers [26].

From the above research trials by other researchers, there is also the possibility to introduce multi-layers during the production process using co-extrusion; however, the final cost for this manufacturing process is significant [27,28]. For instance, as a substitute for HDPE, POK can be used in fuel delivery systems, which exhibits good barrier properties. It also has excellent impact strength, mechanical and thermal properties, chemical resistance, and a low permeation to gases and liquids [29,30,31,32]. POK is polar and has strong inter-molecular interactions between the neighboring polymer chains resulting in low diffusion [33]. These polar and non-polar groups determine the permeation of hydrocarbons into polymers and the changes in thermal and mechanical properties.

Over the last 40 years a lot of research has been focused on polymer interaction with petroleum fuels and the effects of these fuels on polymer properties. However, no papers have been published to address HDPE or POK interaction with BT. In this paper, the physical and chemical properties of POK are compared to HDPE in the presence of hydrogen and BT-containing fuel. Storing this chemically bonded hydrogen in polymeric tanks for a long time can also result in the absorption and diffusion of LOHCs into the plastic over time. Eventually, this could lead to stress cracking, degradation, and a reduction in the mechanical properties of the polymer. Hence, it is important to investigate the aging properties and choose the right material to store LOHCs for a longer duration.

## 2. Materials and Methods

### 2.1. Materials

In this study, two different grades of semi-crystalline materials were selected: HDPE (Lupolen 4261 A IM BD) with a density of 0.940 g/cm^3^ was provided by LyondellBasell, Frankfurt, Germany. Poketone (M330F) with a density of 1.24 g/cm^3^ was obtained from Hyosung, Seoul, Korea. Both are special grades of polymers and have good barrier properties and chemical resistance to fuels in automotive applications. The materials used to investigate are virgin polymers, and no formulations were used during this work. 

### 2.2. Sample Preparation

**Figure 1:**
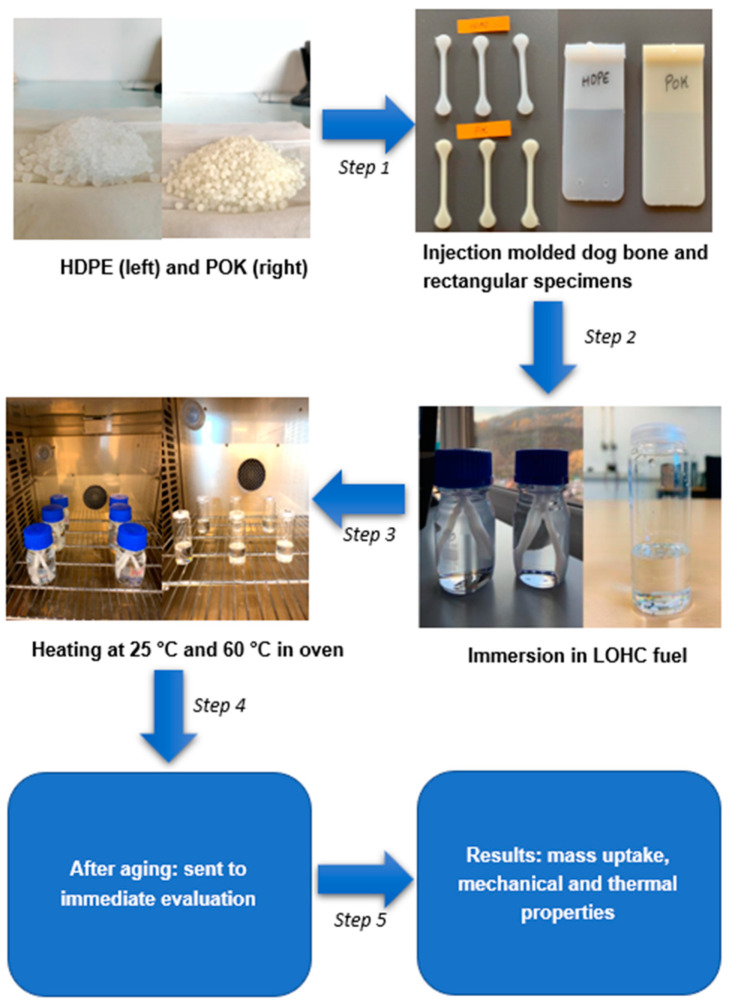
The step-by-step process of injection-molded HDPE and POK samples aged in LOHC fuel in closed chamber.

As shown in Figure 1, in the first step, HDPE and POK pellets were pre-dried in a dryer (DP615, Piovan S.p.A, Venice, Italy) for 3 h before being placed into an injection molding machine. Two different mold plates were used to produce dumbbell and rectangular specimens. Injection-molded cylindrical dumbbell specimens with length = 88 mm and diameter = 5 mm and rectangular plates with length = 138 mm, width = 74 mm, and thickness = 1 mm (as shown in Figure 1) were produced using two different Arburg injection molding machines with an injection speed of 30 and 25 cm^3^/s. For HDPE the nozzle temperature was set to 260 °C, and for POK to 250 °C; both mold temperatures were maintained at 60 °C. The rectangular plates were punched with a hydraulic press of 4 mm diameter to obtain circular samples. They were further used to perform gravimetric analysis, DSC, TGA, and FTIR measurements.

Following that, LOHC fuel was put into 100 mL glass tubes, which were entirely filled, and cylindrical dumbbell specimens were dipped to evaluate their mechanical properties. On the other hand, small glass vials were half filled, and circular samples were placed for thermal evaluation. Later on, the samples were aged in a closed oven for up to 500 h at a constant temperature of 25 and 60 °C, respectively. Then, the completely aged samples were removed from the LOHC fuel and wiped with tissues to remove fuel content on the polymer surface to make sure no error values can be recorded during testing. Finally, the samples were sent for immediate evaluation to investigate the test results, which are mentioned in Section 3. In this research, a total of three replicates were tested in each experimental trial.

### 2.3. Type of Fuel Used

In this study an LOHC with similar properties to diesel was used, with a flash point of 125–180 °C. The advantage of this fuel is that it can be stored under ambient conditions, it is non-flammable, safe, and can be used in automotive fuel tank applications. This LOHC was delivered from Hydrogenious LOHC Technologies GmbH, Erlangen, Germany. 

### 2.4. Ageing Procedure

The dumbbell-shaped specimens of HDPE and POK samples were immersed in 100 mL sealed glass containers which were filled with LOHC fluids at 25 °C and 60 °C for up to 500 h. At regular time intervals, samples were removed from the glass bottles to measure weight changes. Likewise, the circular disk-shaped specimens for DSC and TGA experiments were placed in small glass vials. Then, 10 mL of LOHC was poured into a 50 mL headspace crimp vial and the caps were sealed with tape around them.

### 2.5. Sorption Experiments

The weight gain or weight loss of samples after exposure to LOHC solution were studied using gravimetric analysis. At 60 °C, and also in ambient conditions (25 °C), three replicates of each polymer were weighed in the dry stage and immersed in LOHC solution. Later on, each sample was taken out of the LOHC at regular intervals and weighed. The weight measurement values were recorded on a laboratory electronic microbalance (type M2P, Sartorius AG, Goettingen, Germany). The relative LOHC uptake ($M_{t})$ was calculated using Equation (1):

(1) $$M_{t}=\frac{W_{w}-W_{d}}{W_{d}}$$

$W_{w}$ is the weight of the specimen after exposing to LOHC; $W_{d}$ is the initial weight of the sample before immersion; and $M_{t}$ is the relative mass uptake with respect to time *t.*

### 2.6. Diffusion Coefficient

There are several ways to determine the diffusion of chemicals in polymer materials. Using Fick’s law and assuming a single-phase diffusion process is one of the standard methods for describing molecular diffusion in polymers. As time passes, the relative mass of the absorbed phase will grow linearly. The diffusion coefficient (*D*) is determined using the following equation [34].

(2) $$\frac{M_{t}}{M_{m}}=\frac{4}{\sqrt{\pi }}\sqrt{\frac{Dt}{h^{2}}}\;\;$$

$M_{t}$ is the LOHC uptake, $M_{m}$ is the equilibrium concentration of the corresponding curves, *t* is the immersion time and *h* is the thickness of the specimen.

### 2.7. Differential Scanning Calorimetry (DSC)

In order to characterize the changes in the melting behavior of both materials, HDPE and POK were examined using a DSC1 (Mettler-Toledo, Greifensee, Switzerland, Software STARe V16.30a). The samples, weighing 13 mg (HDPE) and 20 mg (POK), were placed in 40 μL aluminum pans. In a nitrogen atmosphere with a gas flow rate of 50 mL min^−1^, the samples were heated from 20 to 250 °C at a rate of 10 K min^−1^. Before the initial tests, the samples were first removed from the LOHC, and cleaned with dry tissue paper. 

To determine the degree of crystallinity of HDPE and POK, the melting enthalpy is divided by the theoretical melting enthalpy of the 100% crystalline phase (HDPE: 293 J/g [35]; POK: 227 J/g [36]). Therefore, the degree of crystallinity (*X_c_*) was calculated using the following equation [27].

(3) $${\;\;\;\;\;\;\;\;\;\;\;\;\;\;\;\;\;\;\;X}_{c}=\frac{{∆H}_{m}}{\Delta H_{m}^{100\%}}\cdot 100\%$$

### 2.8. Thermal Gravimetric Analyzer (TGA)

To determine the fuel uptake of HDPE and POK, the samples were measured using TG/DSC 3 (Mettler-Toledo, Greifensee, Switzerland). Samples weighing 13 mg (HDPE) and 20 mg (POK) were placed in 70 μL alumina crucibles. With a heating rate of 10 K min^−1^ and a nitrogen gas flow rate of 50 mL min^−1^, TG curves were obtained between 25 and 600 °C under N_2_. In addition, further measurement values were also recorded from 600 to 800 °C in an oxygen environment with a gas flow rate of 50 mL min ^−1^.

### 2.9. Mechanical Testing

The dumbbell specimens for the tensile tests were conditioned by immersion in LOHC fuel up to saturation. The measurements were performed on a Zwick Roell Universal Testing Machine at 25 ± 1 °C with a test speed of 50 mm/min. The gauge length of these specimens was 50 mm. 

### 2.10. Fourier-Transform Infrared Spectroscopy (FTIR)

The infrared spectra were assessed using the Bruker Vertex 70 system (Rosenheim, Germany), which was furnished with an integrated single reflection crystal made of diamond–germanium for ATR measurements. ATR analyses were performed on the surfaces of distinct tablet samples, each exposed to the LOHC for varying durations.

## 3. Results and Discussions

In the pursuit of exploring the viability of utilizing HDPE and POK as potential candidates for long-term LOHC tank systems, a comprehensive investigation was under-taken. This study involved subjecting both HDPE and POK to prolonged exposure at elevated temperatures within the LOHC environment. The testing regiment encompassed a series of crucial evaluations, including tensile testing, fuel uptake analysis, diffusion studies, melt behavior assessments, FTIR spectroscopy, and measurements of thermal stability. In this section, we present a detailed exposition of the obtained results, which contribute significantly to the broader field of hydrogen storage research and facilitate the development of more efficient and sustainable energy solutions. 

### 3.1. Fuel Uptake

#### Chemical Effects

**Figure 2:**
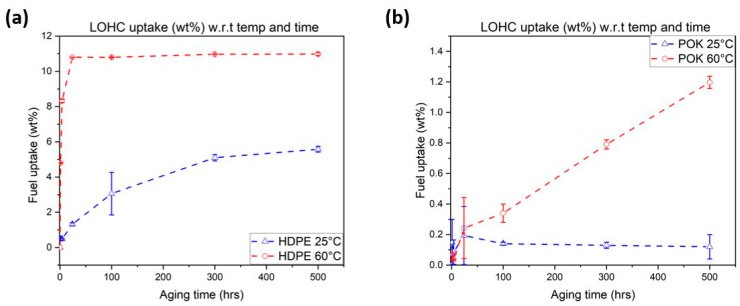
A study of the fuel uptake in injection-molded (**a**) HDPE and (**b**) POK samples which are exposed to air and LOHC fuel in small glass vials. The curves represent mean values along with the standard deviation based on 3 measurements.

Figure 2a,b depict the weight changes in the HDPE and POK samples after immersion at 25 °C and 60 °C with respect to time. 

After exposure to 500 h in LOHC, HDPE reached a plateau level at 60 °C. It is also noticed that if the temperature increases, the absorption rate of HDPE also increases because, at elevated temperatures, the polymer chains are more mobile, resulting in an increase in the free volume of thermoplastics, which allows molecules to diffuse more easily [37]. At 25 °C the absorption rate is significantly lower because, at low temperatures, atoms are more densely packed in amorphous regions, and thus the absorption rate is very low for chemical fluids into polymers. In addition, the fuel uptake percentage of HDPE can be compared with commercial fuels using the work carried out by Libia et al. [38] because the physical properties of these fuels are similar to those of LOHC [19]. In their work they found that the fuel uptake in HDPE at room temperature (25 °C) was about 5.0–5.3 weight % which is very similar to the results obtained with HDPE aged in LOHC fuel. 

Aged samples of POK showed a significantly lower increase in weight with increasing temperature when compared to HDPE. According to Figure 2b, there was an initial drop in mass at the beginning of the exposure time for both 25 and 60 °C. This drop may be attributed to volatilities of residual water or organic additives which might evaporate over time, but there was no degradation caused during these trials. To ensure the material was not dissolved in LOHC, gas chromatography was conducted, but no traces of olefins or carbon monoxide (CO) were found. Additionally, a centrifuge device (Heraeus Labofuge 300) from Thermo Electron (Waltham, MA, USA) was used by inserting small glass tubes in the centrifugal unit and rotating them at 2000 rpm for 300 s. The goal was to investigate the existence of solid particles. However, no polymer particles were found during this process. 

POK is generally polar and contains carbonyl groups which are electronegative. These groups attract electrons away from neighboring carbon atoms, enhancing the intermolecular attraction of the polymer chains [29]. As a result, POK exhibits low absorption of liquids and gases compared to HDPE. At 60 °C these samples did not reach the equilibrium state and grew linearly with time. Therefore, POK could be compared with the work conducted by Zhao et al. [24]. In this work, they investigated the absorption rate of diesel and biodiesels in two different grades of polyamides (which was 1.6 and 2.0 wt%). At 700 h none of these fuels reached an equilibrium state because polyamides contain amide groups and are also polar in nature and this makes the diffusion process slow during the ageing process.

Moreover, H12-BT also contains saturated aromatic hydrocarbons like diesel, which are non-polar in nature and that tend to diffuse into polymers. 

Comparing both graphs, the fuel uptake in HDPE increased rapidly i.e., ~5.5 wt.% and ~11 wt.% under isothermal conditions (25 °C and 60 °C) contrary to POK which was significantly lower at 0.2 wt.% and 1.1 wt.%. at 500 h.

The diffusion coefficient is calculated from Equation (2) using the initial stages of the sorption phase. It is clear that HDPE is more sensitive to LOHC fuel. As the aging temperature increased, the rate of diffusion also increased from 1.2 × 10^−7^ to 1.64 × 10^−7^ cm^2^ s^−1^, which further obeys the Fickian diffusion model. In the case of POK, diffusion is less at 25 °C with 5.6 × 10^−8^ cm^2^ s^−1^, showing high resistance to LOHC fuel. As it did not reach an equilibrium state at 60 °C, this follows the non-Fickian diffusion model, and no diffusion coefficient could be determined for POK exposed to 60 °C. Table 1 shows the measured values of the diffusion coefficients.

### 3.2. Annealing Effects and Crystallinity

**Figure 3:**
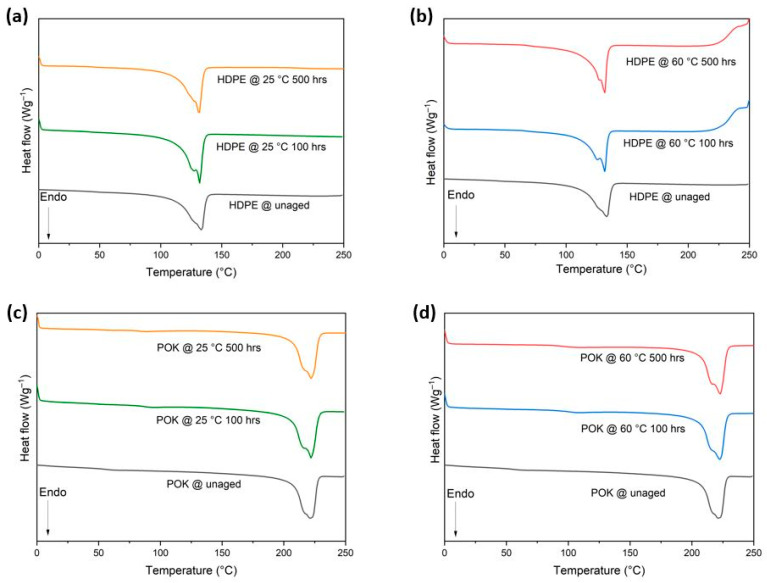
(**a**,**b**) DSC first heating curves for unaged and aged samples of HDPE in LOHC fuel at 25 °C and 60 °C measured for 100 h and 500 h; (**c**,**d**) DSC first heating curves for POK samples on same aging conditions as HDPE (all curves are based on mean values of three measurements).

The melting behavior of the samples was determined using DSC before and after exposure to LOHC fuel. The melting peak temperature (T_m_) and enthalpy (ΔH_m_) values of the first heating scan are shown in Table 2. A single melting peak at 132.6 °C (HDPE) and 220.9 °C (POK) was recorded in unaged samples (shown in Figure 3). Note that the ΔH_m_ of HDPE increased in aged samples compared to unaged. Likewise, enthalpy also increased in POK aging samples which were aged for 100 h to 500 h.

Figure 3a–d depicts the first heating cycle of HDPE and POK thermograms. For these measurements, all the samples underwent chemical oxidation in the oven. As a result of aging at 60 °C, the melting endotherms of HDPE samples showed a slight decrease in melting temperature with an average peak value of 130.5 °C (after aging for 500 h) when compared to samples aged at 25 °C, which showed an average peak value of 131.4 °C. Furthermore, as the aging temperature increased, (60 °C) the melting temperature of POK also increased with a melting peak of 222.6 °C at 500 h. The samples aged at 25 °C were almost similar to samples that were aged at 60 °C with a melting temperature of 221.8 °C. 

As shown in Table 2, the rise in enthalpy ΔH_m_ from the first scan in POK from 74.9 J/g (before immersion) to 81.9 J/g and 84 J/g at 25 °C and 60 °C for 500 h (after immersion) in LOHC fuel is due to the strong intermolecular bonding (spaces reduce between polar groups), which leads to an increase in melting point [39]. Thus, from the below DSC graphs, it can be concluded that with increasing aging time and aging temperature the melting temperature of HDPE decreased slightly, whereas, in the case of POK, the melting temperature increased after aging in LOHC fuel.

From Table 3 it is observed that HDPE crystallinity percentage decreased during the aging process at 60 °C and POK crystallinity percentage (X_c_) increased slightly with increasing aging temperature. 

In HDPE, at 25 °C (500 h) the degree of crystallinity is reduced a little. This phenomenon occurs due to the fact that fuels like H12-BT can penetrate into the polymers in the amorphous region and disrupt the intermolecular forces in polymer chains, resulting in a reduction in crystallinity percentage over time from 100 to 500 h [40]. In addition to this, HDPE also exhibited a higher absorption rate compared to POK. This absorbed LOHC fuel is trapped in between the polymer chains and acts as a plasticizer that could lead to reduction in the crystallinity of the material [38]. 

In the case of POK, a minor increase in crystallinity was observed, which was ~5% after aging for longer exposure times at 25 °C and 60 °C when compared to the unaged samples. This could be due to the interaction of polar carbonyl groups within the LOHC fuel and the rearrangement of polymer chains at elevated temperatures. In addition to this, for all the unaged and aged samples a small shoulder appears before the melting peak which is caused by annealing effects at high temperatures. 

### 3.3. Thermogravimetric Analysis (TGA)

**Figure 4:**
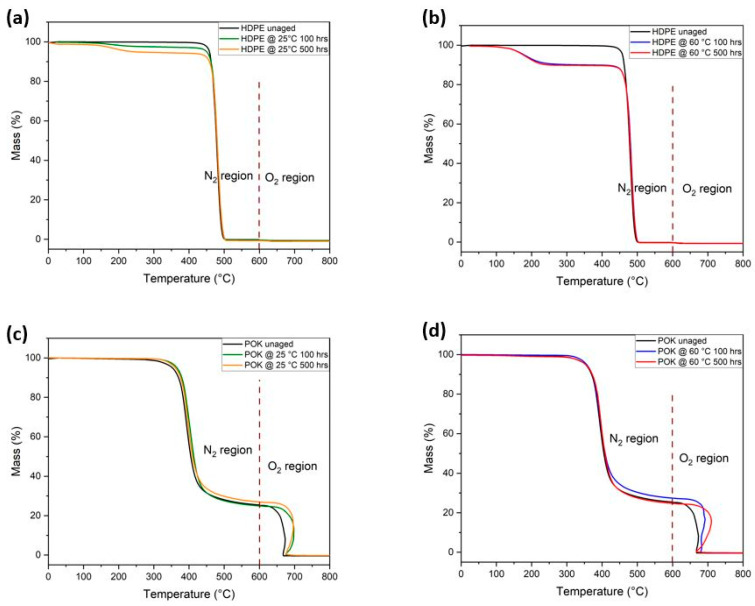
(**a**,**b**) TGA thermograms of unaged and aged injection-molded HDPE samples at 25 and 60 °C; (**c**,**d**) are the TGA thermograms of unaged and aged POK samples at 25 and 60 °C (all curves are based on mean values of three measurements).

According to Figure 4a,b, HDPE exhibited mass losses between 100 and 300 °C before decomposition, which occurred at approximately 500 °C. This initial mass loss in the aged samples can be attributed to the evaporation of absorbed fuel, which reflects the fuel uptake measurements. As HDPE is exposed to LOHC fuel for 500 h under ambient conditions (25 °C), degradation is slightly faster than the sample aged at 100 h which can be seen in Figure 4a. In addition to this, the degradation caused by the mass loss of samples aged in LOHC fuel at 60 °C for 500 h is much faster than the samples aged for 500 h at 25 °C.

Figure 4c,d shows POK thermograms from the TGA, indicating a two-step process before complete degradation occurs. As the absorption rate is very low in POK, no mass loss occurred below 200 °C. All the samples started to lose mass in between 200 °C and 400 °C. In addition to that, only 75% of the polymer broke down under a nitrogen atmosphere, where the ketone groups being eliminated during the first step of the process. The remaining 25% of the degradation occurred in the second step under the oxygen atmosphere. This particular step plays a crucial role in the analytical process, allowing us to discern and quantify the presence of non-hydrocarbon materials within the samples. It is also noticed that the rate of degradation of POK in the first step is faster than the second step which was also observed by Al-Muaikel et al. [41]. Similar to HDPE, POK also lost mass slightly faster for samples aged at 60 °C when compared to samples aged at 25 °C.

Overall, it is worth noting that, based on the weight loss from volatile compounds, POK demonstrates greater inertness to LOHC fuel compared to HDPE. However, it is shown that the POK’s drawback lies in its lower thermal stability, which needs to be enhanced through the use of stabilizers.

### 3.4. Mechanical Testing

**Figure 5:**
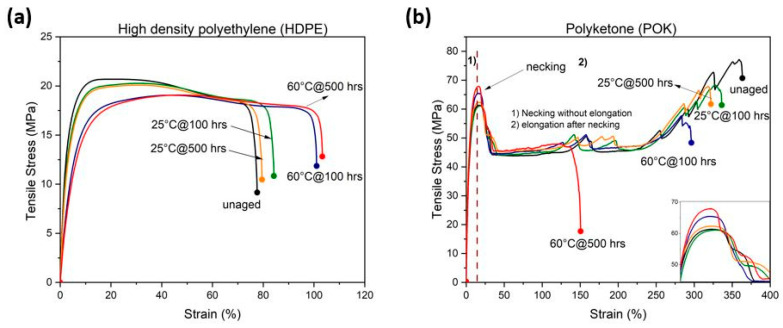
The stress–strain curves of aged and unaged samples of (**a**) HDPE and (**b**) POK in a closed oven are measured after immersion for up to 500 h in LOHC fuel. The inset in (**b**) shows the magnified detail of the stress peaks in the necking region (all curves are based on mean values of three measurements).

Figure 5a illustrates the stress–strain curves of injection molded HDPE samples immersed in LOHC fuel which are aged in a closed oven at 25 °C and 60 °C. It shows that the maximum stress (σ) of all the aged polymers decreased during exposure to LOHC with respect to time when compared to unaged samples, and this is due to the decrease in crystallinity. On the other hand, the strain at break is increased by immersing samples for a longer time in the LOHC fuel. A distinct difference in the elongation at break was also observed between aged and unaged samples. The strain percentage of the aged sample increased from 79% to 84% at 25 °C over time; at 60 °C, the sample increased from 101% to 103%. But there is a huge difference between the aged samples at 60 °C and the unaged samples, where the unaged sample remained at 77%. This increased strain percentage was due to the higher fuel concentration in the polymers which leads to a plasticizing effect that can soften the material over time. 

According to Figure 5b, POK shows a rise in the maximum stress (σ) in the initial region of the curve after the aging process. At higher strains, the tensile stress of the POK samples decreases gradually due to strain hardening. It is also noticed that the strain at break of aged samples almost remained constant at 25 °C over time, whereas at the elevated temperature of 60 °C, a sharp decline in strain at break was observed from about 300% to 150% which could be due to the increased crystallinity and a reduction in the mobility of the polymer chains that makes the material less flexible and decreases the strain at break. In addition, due to the aging at high temperatures for 500 h, the intermolecular forces in POK may improve and strengthen the material, leading to a slight increase in tensile stress from 60 MPa to 67 MPa. In Table 4, the E-modules of HDPE and POK are listed along with the strain at break percentage.

The above results indicate that the exposure temperature of the LOHC strongly influences the tensile behavior of HDPE and POK. The POK aged in LOHC showed higher tensile properties and a drop in elongation, whereas, in the case of HDPE, the elongation at break percentage increased but tensile properties decreased due to the plasticization effect.

### 3.5. FTIR Spectrum

**Figure 6:**
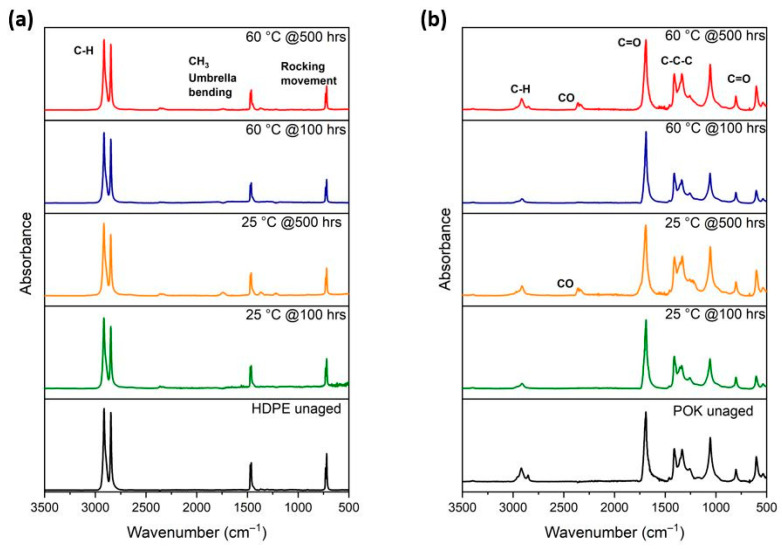
(**a**,**b**) are the FTIR spectra of the unaged and aged (**a**) HDPE and (**b**) POK at 25 and 60 °C for 100 and 500 h.

Figure 6a,b contrasts the FTIR spectra of HDPE and POK that are displayed for before and after being immersed in LOHC until 500 h at both 25 and 60 °C. All the spectra exhibit characteristic IR absorbance peaks commonly associated with HDPE and POK. For HDPE, the peaks at 2913 and 2847 cm^−1^ correspond to asymmetric and symmetric C-H bond stretching, respectively. Additionally, a peak at 1461 and 1463 cm^−1^ indicates the presence of CH_3_ umbrella bending mode, while the peak at 719 and 720 cm^−1^ signifies a rocking movement.

In the case of POK, a distinct peak at 1687 cm^−1^ indicates the presence of carbonyl groups. The methylene groups in polyketones contribute to a broad peak in the range of 2800–3000 cm^−1^, and there are also additional peaks around 1300 cm^−1^ corresponding to stretching vibrations of carbon–carbon–carbon (C-C-C) bonds. Peaks at 600 and 800 cm^−1^ represent C=O bond stretching. By comparing the immersed samples to the non-immersed one, it can be concluded that no noticeable chemical changes occurred as a result of LOHC absorption in the polymers. Despite the increase in temperature, there is also no discernible difference in the FTIR spectra of HDPE and POK.

## 4. Conclusions

This study compared the aging behavior of HDPE and POK that are exposed to LOHCs (at ambient conditions and at 60 °C). HDPE, as a non-polar polymer, showed a greater fuel uptake compared to POK after 3 weeks. However, an initial mass loss was observed in POK below 100 h at 60 °C due to the evaporation of volatiles. Nevertheless, the POK samples did not reach a plateau level like HDPE, and therefore, more immersion tests need to be performed. From mass uptake measurements, the diffusion of chemicals into polymers was calculated. The diffusion coefficient increased from 1.25 × 10^−7^ at 25 °C to 1.64 × 10^−7^ cm^2^ s^−1^ in HDPE at 60 °C due to the weak bonds in the polymer chain, whereas, in POK, the rate of diffusion was less with 5.62 × 10^−8^ cm^2^ s^−1^ at 25 °C.

Fuel uptake, plasticization effect, and crystallinity led to differences in tensile strengths of HDPE and POK. Due to the presence of non-polar groups the absorption rate of HDPE samples immersed in LOHC fuel was increased. The LOHC fuel used in this work contains hydrocarbons which act as plasticizers. Fuels like these can penetrate and break down the polymer chains and increase the free volume between them, which results in a decrease in crystallinity and tensile strength and increased flexibility. Thus, HDPE lost more strength and the elongation at break percentage increased due to the plasticization effect. 

In the case of POK, LOHC fuel exposure may cause polymer chain restructuring. The interaction with the fuel can result in the creation of highly ordered and crystalline regions within the polymer matrix, improving its overall crystallinity and tensile strength and making the material more rigid. Therefore, the elongation at break percentage was reduced in POK as the aging temperature increased from 25 °C to 60 °C, but the tensile strength increased gradually after aging for 500 h. This could be due to the formation or enhancement of crosslinking within the polymer structure. 

Furthermore, the FTIR results indicated no significant chemical changes in both materials. After the long experimental trials neither of the wet specimens developed discoloration or cracks.

Therefore, in order to store fuels like LOHC for a longer time, not only at room temperature (25 °C) but even in high temperatures (60 °C), POK could be considered as an alternative choice to store and transport hydrogen due to its excellent barrier properties and mechanical properties when compared to HDPE, because materials like HDPE are less suitable for applications that require a high mechanical strength and dimensional stability.

## Figures and Tables

**Table 1:** Diffusion coefficient *D* and equilibrium concentration $M_{m}$ of LOHC fuel in HDPE and POK.

Temperature (°C)	HDPE (*D* in (cm^2^ s^−1^))	POK (*D* in (cm^2^ s^−1^))	$M_{m}$ (HDPE/POK) (-)
25	1.25 × 10^−7^	5.6 × 10^−8^	0.0557/0.0014
60	1.64 × 10^−7^	-	0.1098/-

**Table 2:** Melting temperature (T_m_) and melting enthalpy (ΔH_m_) of HDPE and POK samples aged in LOHC fuel for 100 and 500 h at 25 and 60 °C.

Sample	T_m_ (°C) 1st Heating	ΔH_m_ (J/g) 1st Heating
Unaged @HDPE	132.6 ± 1.4	135.6 ± 1.6
HDPE @25 °C/100 h	131.9 ± 0.3	144.9 ± 2.5
HDPE @25 °C/500 h	131.4 ± 0.1	142.4 ± 3.1
HDPE @60 °C/100 h	130.6 ± 0.7	122.1 ± 1.2
HDPE @60 °C/500 h	130.5 ± 1.2	137.2 ± 1.0
Unaged @POK	220.9 ± 0.7	74.9 ± 0.6
POK @25 °C/100 h	221.8 ± 0.1	82.6 ± 1.3
POK @25 °C/500 h	221.8 ± 0.2	81.9 ± 1.4
POK @60 °C/100 h	222.3 ± 0.6	83.6 ± 0.8
POK @60 °C/500 h	222.6 ± 0.4	84.0 ± 2.0

**Table 3:** Crystallinity (X_c_) of aged and unaged samples of HDPE and POK obtained from DSC measurements as a function of aging times that are obtained using Equation (3).

Aging Time (h)	Material 1	Crystallinity (%) (1st Heating)	Material 2	Crystallinity (%) (1st Heating)
unaged	HDPE @25 °C	46.3 ± 0.3	POK @25 °C	33.0 ± 0.2
100		49.5 ± 0.8		36.4 ± 0.6
500		48.6 ± 0.9		36.1 ± 0.6
100	HDPE @60 °C	41.7 ± 0.4	POK @60 °C	36.8 ± 0.4
500		46.8 ± 0.3		37.0 ± 1.0

**Table 4:** Youngs modulus E and strain at break percentage of HDPE and POK are defined below.

Aging Time (h)	Oven Temperature (°C)	E-Modulus (GPa) (HDPE)	E-Modulus (GPa) (POK)	Strain (%) (HDPE)	Strain (%) (POK)
Unaged	25	0.61 ± 0.01	1.96 ± 0.20	78 ± 3.9	363 ± 29
Aged @ 100	25	0.55 ± 0.01	1.70 ± 0.01	84 ± 7.7	336 ± 43
Aged @ 500	25	0.53 ± 0.01	1.72 ± 0.04	80 ± 3.1	322 ± 40
Aged @ 100	60	0.30 ± 0.01	1.56 ± 0.09	101 ± 2	296 ± 25
Aged @ 500	60	0.27 ± 0.01	1.63 ± 0.01	103 ± 8.4	151 ± 33

## Data Availability

The data presented in this study are available on request from the corresponding author.
